# The economic benefits of malaria elimination: do they include increases in tourism?

**DOI:** 10.1186/1475-2875-11-244

**Published:** 2012-07-28

**Authors:** Sepideh Modrek, Jenny Liu, Roland Gosling, Richard GA Feachem

**Affiliations:** 1 Global Health Group, University of California, San Francisco, CA, USA

**Keywords:** Malaria elimination, Tourism, Economic benefits

## Abstract

**Background:**

Policy makers have speculated that one of the economic benefits of malaria elimination includes increases in foreign direct investment, particularly tourism.

**Methods:**

This study examines the empirical relationship between the demand for travel and malaria cases in two countries with large tourism industries around the time in which they carried out malaria-elimination campaigns. In Mauritius, this analysis examines historical, yearly tourist arrivals and malaria cases from 1978–1999, accounting for the background secular trend of increasing international travel. In Dominican Republic, a country embarking upon malaria elimination, it employs a time-series analysis of the monthly, international tourist arrivals from 1998–2010 to determine whether the timing of significant deviations in tourist arrivals coincides with malaria outbreaks.

**Results:**

While naïve relationships exist in both cases, the results show that the relationships between tourist arrivals and malaria cases are relatively weak and statistically insignificant once secular confounders are accounted for.

**Conclusions:**

This suggests that any economic benefits from tourism that may be derived from actively pursuing elimination in countries that have high tourism potential are likely to be small when measured at a national level. Rather, tourism benefits are likely to be experienced with greater impact in more concentrated tourist areas within countries, and future studies should seek to assess these relationships at a regional or local level.

## Background

Assessing the economic benefits of eliminating malaria is a challenging topic, but one that has received some attention [1-4]. The economic gains of moving from high malaria burden to low burden are well documented, including productivity gains such as increased human capital and increased productivity of factors of production, such as land or capital [5,6]. While there are likely to be sizeable gains in moving from no malaria control activities to large-scale control activities, it is unclear how large these gains may be and where economic gains may be found when moving from a state of controlled, low endemic malaria to malaria elimination, i.e. essentially moving from very low burden to no burden of malaria.

The clearest argument for pursuing malaria elimination focuses on the global and regional public good of removing the threat of a deadly and epidemic-prone disease [7,8]. Other broad economic benefits have been postulated, such as sustained gains in human capital which may alter the trajectory of investment decisions of individuals, foreign investors, and local firms that together change the productivity of the country and region after elimination [2,3]. The arguments generally suggest that there are “take-off” effects or changes in trajectories once a country commits to malaria elimination, but these effects have been difficult to quantify due to limited available data.

Many have hypothesized that the take-off benefits associated with the decision to eliminate may largely manifest in foreign direct investment (FDI), including tourism [3]. However, to date, there is little empirical evidence to support these claims. The anecdotal evidence commonly referred to in malaria-elimination debates should be evaluated with greater emphasis on context and generalizability, as well as greater scrutiny of the quality of analytical rigour used to assess the validity of these statements.

Only one quantitative study, which focused on the broad determinates of tourism in 43 African countries, examined the impact of malaria rates on tourism [9]. This study used a variety of methods (ordinary least squares, random effects regressions, and generalized methods of moments) applied to country-level panel data (1996–2002), and found inconsistent associations between tourism and malaria rates (measured in 1994 and used as a proxy for health risk). The most reliable estimates in the study (using Arellano-Bond generalized method of moments) suggest that malaria was only marginally related to tourism from Europe with an estimated magnitude that is relatively small. The lack of consistent findings may be related to the inclusion of many countries with lower tourism potential as well as using only one static measure of malaria burden. Rather, the tourism industry is likely affected by many factors, such as having unique geographical and climatic features or historical legacies, which contribute to the attractiveness of the locality. Thus, tourism benefits are likely to be concentrated in specific countries that have yet to fully develop their tourism potential.

This analysis takes a closer look at the secular relationship between tourism and malaria elimination in two countries where tourism revenues comprise a significant portion of the economy: Mauritius and the Dominican Republic. In the former, the relationship between the numbers of tourists and reported malaria cases taking into consideration global trends in travel is examined. In the latter, the analysis focuses on the observed trends in monthly international tourist arrivals, looking for variations from the expected versus the actual numbers of tourists visiting the Dominican Republic.

Mauritius provides a particularly interesting case to study potential benefits of malaria elimination for two reasons. First, Mauritius achieved elimination of local transmission in 1969, but then experienced a re-emergence of malaria that required a second elimination campaign. At the time of the second elimination campaign, the tourism industry was still in its infancy, providing an opportunity to examine the long-term association with growth in the tourism industry: currently, about 31.7% of Mauritius’ GDP comes from the tourism industry [10].

This study also explores the relationship between tourism and malaria in a country that has recently declared a goal of elimination, the Dominican Republic (DR), in order to investigate the immediate effects of malaria outbreaks compared to other singular disruptions to the tourism industry, such as natural disasters. The DR has been pursuing elimination since late 2008 and is a particularly interesting case because of its location on Hispaniola Island. Over 97% of malaria cases on Hispaniola occur in DR’s neighbour, Haiti, which contributes to a large number of imported cases from migrant workers in DR [11]. Meanwhile, the tourism industry in DR has grown rapidly over the last 30 years, now accounting for 17.7% of GDP [12].

## Methods

### Mauritius

Historical, national level data are compiled from three sources. The main outcome variable, the yearly number of tourist arrivals into Mauritius from 1974–2005, is sourced from the Central Bank of Mauritius. Tourism information on international passengers travelling through the United Kingdom (UK) (UK; 1978–1999) is drawn from a report from the UK Parliamentary Office of Science and Technology. This measure includes transit passengers from other origins passing through all UK airports, including all five of London’s international airports. This measure is used as a control for the supply of international tourists because London’s airports, particularly London Heathrow and London Gatwick, have been among the busiest airports for international travel [13]. London Heathrow has consistently ranked first in terms of the number of international travellers. Likewise, London Gatwick has historically ranked in the top 10. Many travellers from other origins have historically passed through London’s airports and thus are accounted for in this measure [14]. The primary independent variable, the historical incidence of malaria and the malaria-elimination campaign years, is obtained from Tatarsky et al. [15].

The following log-log linear regression model was used to estimate the incremental percentage change in tourism with respect to changes in malaria cases:

(1) $$\ln\left(Tourists\right)=\alpha +\beta (\ln\left(Malaria\;Cases\right)+\gamma (\ln\left(UK\;Travellers\right)+\epsilon$$

the interpretation of the estimated coefficient, β, is the elasticity of malaria cases with respect to tourists arriving in Mauritius, and γ is the elasticity of UK international travellers with respect to tourists arriving in Mauritius. The estimated models are meant to be illustrative, and not necessarily causal as time-series data requires that we consider the autocorrelation structures inherent in the data.

### Dominican Republic

Data for the DR are compiled from two sources. Information on the number of malaria cases comes from the 2010 World Malaria Report [16]. The number of tourist arrivals by month is obtained from the Central Bank of the Dominican Republic’s website [17]. Information on the occurrence of natural disasters is downloaded from the International Disasters Database [18].

Time-series methods were employed to determine significant deviations from expected inflows based on the monthly, international tourist arrivals data to the DR. The number of travellers was logged to attain an estimate of the percentage change in tourist arrivals. Univariate, 12-month, seasonally adjusted, autoregressive, integrated moving average models (ARIMA) with no autocorrelation parameters were implemented in Stata (StataCorp, 12, College Station, Texas, USA) to forecast the number of expected arrivals in any given month based on the historical trends in international tourist arrivals. The difference between the observed actual and predicted log of travellers was computed. These deviations were plotted, and deviations outside of the estimated 95% confidence interval were marked.

## Results and discussion

### Mauritius

**Figure 1:**
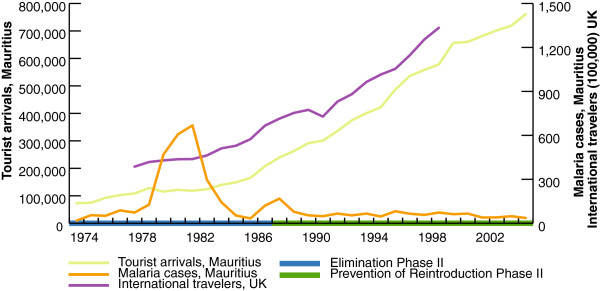
**Secular trends in tourist arrivals and malaria cases in Mauritius, and international travelers passing through the UK.** Souce: UK Parliament and The Central Statistical Ofiice of Mauritius.

This analysis first explores the correlation between tourist arrivals and malaria cases from 1975–2005 in Mauritius with consideration given to the timing of specific malaria elimination efforts (Figure 1). The raw correlation between malaria cases and yearly passenger data is −0.39 (p-val = 0.028). However, these simple correlations ignore broader changes in international travel and vacation patterns driven by world economic growth. During the same period, the supply of air travel increased, prices of air travel fell, and international business activity and leisure time grew. These trends resulted in a dramatic expansion of global air travel. To illustrate this, Figure 1 shows the number of international terminal passengers in UK airports for the same time period. Of particular interest is the end of the second round of elimination activities (1975-1988) which occurs just as the trend in tourist arrivals begins to slope markedly upwards. To the upward change in the slope of tourist arrivals in Mauritius. Thus, at first pass, the directionality of these correlations is consistent with hypothesized benefits of malaria elimination within the “take-off” framework. However, the patterns in international travel mirror the growth in passenger arrivals to Mauritius. Therefore, it is likely that the overall increase in international travel can more readily explain the increase in travellers to Mauritius rather than Mauritius’ efforts to eliminate malaria.

To further understand the magnitudes of these relationships when taking into account international travel, a major confounding factor, the analysis used a simple log-log linear regression (see 1) to examine the relationship between malaria cases and the number of people travelling to Mauritius. In the unadjusted analysis, when the background changes in international air travel are not controlled for, the relationship between malaria cases and tourist arrivals in Mauritius is not significant, even though the magnitude of the coefficient is negative and large (see column 1): a one-fold increase in malaria cases is associated with an 18% decline in tourist arrivals in the same year. However, in the adjusted analysis, when the background increase in international travel is accounted for (see column 2), the estimated effect drops to 2% and is not statistically significant. While this simple regression analysis cannot account for the lagged effects of malaria elimination on tourism (i.e., whether elimination increases future tourism), the fact that much of the relationship between tourist arrivals and malaria cases can largely be explained by overall trends in international travel, and not by reduced malaria risk, suggests that any lagged effects may be minimal. In addition, when using the number of international travellers passing through UK airports as the outcome, the relation with malaria cases is stronger and significant (results not shown)-- an association which cannot be causally related.

**Table 1:** Association of yearly Malaria cases and tourist arrivals in Mauritius

	**Outcome: Ln (Tourist Arrivals)**			
	**Bivariate unadjusted model**		**Adjusted model**	
Ln (Malaria Cases)	−0.18		−0.021	
	[0.15]		[0.024]	
Ln (UK Passengers)			1.47	***
			[0.053]	
Constant	13.03	***	2.88	***
	[0.689]		[0.053]	
N	26		22	
Years	1974-1999		1978-1999	

Note: Each column displays the estimated coefficients and standard errors in brackets from the log-log regression models.

P-value significance level is denoted by *** sig at 1%; ** sig at 5%; * sig at 10%.

Adjusted model accounts for the yearly number of air travellers passing through the UK, which includes transit passengers from other origins passing through Heathrow Airport.

### Dominican Republic

**Figure 2:**
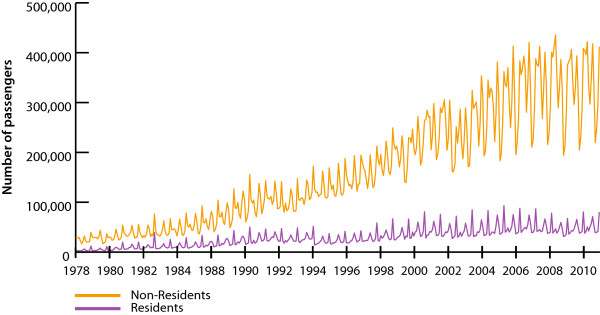
**Total monthly passenger arrivals to the Dominican Republic by air from 1979-2010.** Souce: Central Bank of Dominican Republic.

The study then explores the correlation between the precise timing of tourist arrivals and singular large events that may affect tourism in the Dominican Republic. Figure 2 shows that tourism has been growing in DR long before the national declaration of intent to eliminate malaria. Since the declaration was made in 2008, the time is too short to permit rigorous assessment of the effects of elimination efforts on the tourism industry. However, the effects of malaria outbreaks, which are primarily driven by outbreaks originating in neighbouring Haiti [11], on DR’s tourism industry can be examined. Accordingly, the analysis can identify singular events that have affected tourism, quantify the magnitudes of these events, and place any potential malaria outbreak effects on a relative scale.

**Figure 3:**
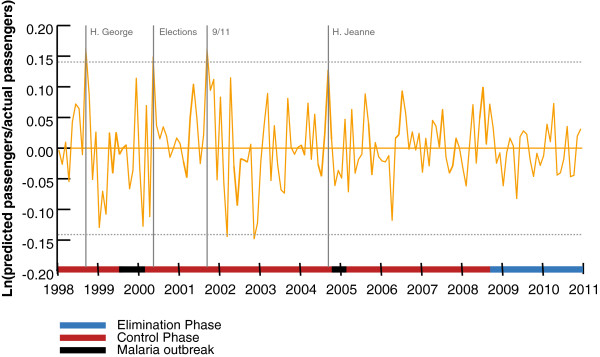
Difference in predicted versus actual non-resident passengers to Dominican Republic from 1998-2010.

With monthly information on the number of non-resident tourist arrivals in DR, it is possible to compare the number of actual arrivals to a model that adjusts for seasonality in order to determine when large deviations from expected travel occur. Figure 3 plots the difference between the expected and actual number of non-resident passenger arrivals by month on a log scale. The log-scale transformation gives the log of the ratio of the actual arrivals divided by the predicted arrivals or the proportionate difference. Focusing only on positive and significant deviations first (significant at the 5% level) where the actual number of passengers is below that which would have been expected, the results suggest that such instances coincide with natural disasters and political events. Figure 3 shows that events such as Hurricane George [September 22 –23, 1998] (347 fatalities and an estimated US$2 billion in damages) or the terrorist events on September 11, 2001 (leading to an unprecedented grounding of flights to and from the USA) are related to decreased tourism in the month of the event. The magnitude of the effects of these two events is about an 18% drop in the number of non-resident passengers in the one to two months immediately after the events. A conservative, back-of-the-envelope calculation suggests that this translates into a US$66 million loss within two months of each event.

These significant deviations do not appear to correlate closely to malaria outbreaks even though there were two during this time period— one in 1999 and another in 2004 [19,20]. In particular, there was a severe outbreak in 2004 in Haiti that precedes the observed rise in malaria cases in DR in 2004. This outbreak event has previously been cited for causing a $200 million loss to DR’s tourism industry [11]. However, the occurrence of this outbreak in 2004 does not coincide with a significant deviation in tourist arrivals. While there is likely to be a correlation between hurricanes and malaria outbreaks, the damage to tourism is likely to be primarily related to the natural disaster itself rather than due to the ensuing outbreak.

## Conclusions

The findings of this study provide little support for a robust association between changes in rates of tourism and either reported malaria cases or reported outbreaks of malaria in two small economies with high tourism revenues and either very low malaria or no malaria burden. The simple models employed in the analysis looked exclusively at tourist arrivals at an aggregated level and did not directly assess tourism revenue or location-specific tourism data. However, using a national-level approach, there appears to be little effect of malaria on tourism in these low-endemic or previously endemic settings. Rather, changes in tourism appear to be more affected by larger and more publicized events, such as political events or natural disasters (e.g., hurricanes). Nonetheless, several caveats should be noted in interpreting these results.

The tourism and malaria burden data from Mauritius include only a small number of yearly observations, which severely limits analytical power. This additionally constrains any possible analysis of lag structures. While it may be possible to find a lagged relationship result with a stronger correlation between the tourist arrivals data and malaria data, the broader issue of controlling for confounding due to background international travel is critical to understanding what factors beyond malaria that may be driving tourism to Mauritius.

In the case of the Dominican Republic, the existence of more refined, monthly data presents a distinct advantage enabling deeper statistical analysis. The analysis uses seasonally adjusted ARIMA models because they can account for the expected fluctuations in the data. Indeed, the model does capture events that are expected *a priori* to affect tourism (i.e., the terrorist attacks of 11 September, 2001). This model is intrinsically more conservative because it only accounts for fluctuations in successive months and between observations year-on-year and no other possible confounders. Therefore, for an acute event, such as a large hurricane, these models can be very useful for examining impacts. However, if the timing of the event and the tourism reaction to it are not tightly linked in a small window of time, it is more difficult to detect smaller deviations using this method. Still, the magnitudes of tourism losses due to malaria outbreaks that have been reported anecdotally elsewhere are much larger than what seems possible given the magnitude effect sizes for large natural disasters.

Notwithstanding these limitations, the results of this analysis echo those found by Naude et al. [9]. Malaria in low endemic or peri-elimination settings does not appear to be robustly associated with tourism at the national level. One potential reason for this lack of overall association may be related to the strength of the spatial correlation between malaria and tourism activities. For example, in the case of Botswana, the main tourist destination of the Okavango Delta is located in the malaria-endemic region of the country. However, in cases such as Namibia, the main tourist areas, such as Swakopmund and much of the Namibian desert, are located far from malaria-endemic areas. Thus, there could be highly localized tourism benefits to malaria elimination that these national-level studies are not capturing. As countries move forward with elimination and as data systems improve, it may become possible to look at these relationships prospectively taking into consideration location-specific data—at which point, we could undertake analyses that would give a clearer picture of the broader implications on the macroeconomic gains/losses due to malaria elimination.

In addition, this analysis did not compare rival destinations and so could not examine the potential for relative advantages conferred by malaria elimination. Specifically, countries eliminating malaria may see an increase in tourism if their main rival destination is already malaria-free. In the case of Dominican Republic, there are many rival malaria-free destinations in the Caribbean that compete with it for tourists, especially when there is an outbreak of malaria, and no evidence of a significant decrease related to malaria was suggested. However, there are other countries engaged in more direct competition for tourists. For example, Vanuatu, with significant malaria transmission in some islands and near elimination in others, competes with Fiji, which has always been malaria-free, for tourists from Australia and New Zealand. Elimination of malaria in all or some of the islands of Vanuatu, other things being equal, may enhance its competitive advantage relative to Fiji. These specific rival cases deserve further scrutiny, and detailed studies of tourists’ destination decision-making processes would be invaluable in understanding the relative weight tourists put on malaria risk when deciding where to travel.

Nonetheless, this study suggests that moving from low-malaria transmission to no-malaria transmission has had little effect on tourism in Dominican Republic and Mauritius, and the economic benefits of malaria elimination on the growth of the tourism sector were likely small or localized.

## Competing interests

The authors declare they have no competing interests.

## Authors’ contributions

SM and JL conceived the idea. SM gathered the data and conducted the analysis. SM and JL structured and wrote the manuscript. RG and RF participated in the presentation of results and contributed to writing the manuscript. All authors read and approved the final manuscript.
